# CD22-Binding Synthetic Sialosides Regulate B Lymphocyte Proliferation Through CD22 Ligand-Dependent and Independent Pathways, and Enhance Antibody Production in Mice

**DOI:** 10.3389/fimmu.2018.00820

**Published:** 2018-04-19

**Authors:** Naoko Matsubara, Akihiro Imamura, Tatsuya Yonemizu, Chizuru Akatsu, Hongrui Yang, Akiharu Ueki, Natsuki Watanabe, Hajjaj Abdu-Allah, Nobutaka Numoto, Hiromu Takematsu, Shinobu Kitazume, Thomas F. Tedder, Jamey D. Marth, Nobutoshi Ito, Hiromune Ando, Hideharu Ishida, Makoto Kiso, Takeshi Tsubata

**Affiliations:** ^1^Department of Immunology, Medical Research Institute, Tokyo Medical and Dental University, Tokyo, Japan; ^2^Department of Applied Bio-Organic Chemistry, Gifu University, Gifu, Japan; ^3^Department of Structural Biology, Medical Research Institute, Tokyo Medical and Dental University, Tokyo, Japan; ^4^Department of Biological Chemistry, Human Health Sciences, Graduate School of Medicine, Kyoto University, Kyoto, Japan; ^5^RIKEN, Wako, Saitama, Japan; ^6^Department of Immunology, Duke University Medical Center, Durham, NC, United States; ^7^Center for Nanomedicine, University of California, Santa Barbara, CA, United States; ^8^Center for Highly Advanced Integration of Nano and Life Sciences (G-CHAIN), Gifu University, Gifu, Japan

**Keywords:** CD22, glycan ligands, synthetic sialoside, adjuvant, B cell

## Abstract

Sialic acid-binding immunoglobulin-like lectins (Siglecs) are expressed in various immune cells and most of them carry signaling functions. High-affinity synthetic sialoside ligands have been developed for various Siglecs. Therapeutic potentials of the nanoparticles and compounds that contain multiple numbers of these sialosides and other reagents such as toxins and antigens have been demonstrated. However, whether immune responses can be regulated by monomeric sialoside ligands has not yet been known. CD22 (also known as Siglec-2) is an inhibitory molecule preferentially expressed in B lymphocytes (B cells) and is constitutively bound and functionally regulated by α2,6 sialic acids expressed on the same cell (cis-ligands). Here, we developed synthetic sialosides GSC718 and GSC839 that bind to CD22 with high affinity (IC_50_ ~100 nM), and inhibit ligand binding of CD22. When B cells are activated by B cell antigen receptor (BCR) ligation, both GSC718 and GSC839 downregulate proliferation of B cells, and this regulation requires both CD22 and α2,6 sialic acids. This result suggests that these sialosides regulate BCR ligation-induced B cell activation by reversing endogenous ligand-mediated regulation of CD22. By contrast, GSC718 and GSC839 augment B cell proliferation induced by TLR ligands or CD40 ligation, and this augmentation requires CD22 but not α2,6 sialic acids. Thus, these sialosides appear to enhance B cell activation by directly suppressing the inhibitory function of CD22 independently of endogenous ligand-mediated regulation. Moreover, GSC839 augments B cell proliferation that depends on both BCR ligation and CD40 ligation as is the case for *in vivo* B cell responses to antigens, and enhanced antibody production to the extent comparable to CpG oligonuleotides or a small amount of alum. Although these known adjuvants induce production of the inflammatory cytokines or accumulation of inflammatory cells, CD22-binding sialosides do not. Thus, synthetic sialosides that bind to CD22 with high-affinity modulate B cell activation through endogenous ligand-dependent and independent pathways, and carry an adjuvant activity without inducing inflammation.

## Introduction

Sialic acid-binding immunoglobulin-like lectins (Siglecs) are type I membrane proteins expressed in various cell types, especially those of immune cells (1). Most of the Siglecs carry signaling function, and each member of the Siglec family is expressed in specific cell types. Therefore, Siglecs are good targets for immune regulation. Although most of the Siglecs bind to sialic acids at their extracellular region, each member shows a distinct specificity for the type and linkage of sialic acid (1). In earlier studies, Kelm et al. generated various sialosides in which the C2, C5, and C9 positions of sialic acid are modified (2), and demonstrated that the sialoside α-9-*N*-(biphenyl-4-carbonyl)-amino-9-deoxy-Neu5Ac (BPC-Neu5Ac) in which the C9 position was modified by a biphenyl markedly improved affinity to human CD22 (4 µM) (3). Introduction of galactose at the C2 position was shown to further improve affinity to human and mouse CD22 (4, 5). Later, we demonstrated that modification of the C9-modified sialoside 9-(4′-hydroxy-4-biphenyl)acetamido-9-deoxy-Neu5Gc (hydroxy-BPAc-Neu5Gc) at C2 position by hydrophobic groups such as benzyl and biphenyl groups augmented affinity to human CD22 by 12-fold (70 nM) and mouse CD22 by 38-fold (100 nM) (6). This finding suggested that introduction of multiple hydrophobic groups in different positions of the sialic acid backbone enhances affinity to CD22. Further modifications in multiple positions generated sialosides with much higher affinity to CD22 (2 nM) (7–10). Efficient synthesis of modified sialosides and selection resulted in development of high-affinity synthetic ligands of other Siglecs, such as Siglec-1 (11), MAG (12), and Siglec-7 (13, 14). Multimers of high-affinity sialoside ligands and nanoparticles carrying these sialosides were developed to target various molecules such as toxins and antigens to Siglecs. Various therapeutic potentials of these multimers and nanoparticles have been demonstrated (15, 16). Targeting of toxins to Siglecs kills lymphoma cells and leukemia cells (17, 18), suggesting therapeutic potential for treatment of the diseases such as leukemia and lymphomas. Targeting lipid antigens to Siglec-1 on antigen-presenting cells enhances activation of NKT cells (19). Moreover, targeting of antigens to CD22 or Siglec-G expressed on B cells co-ligates these Siglecs and the B cell antigen receptor (BCR) specific to the antigens, resulting in deletion of the specific B cells and tolerance to the antigens (20–23). The liposomes displaying Siglec ligands and antigens are called Siglec-engaging tolerance-inducing liposomes (STALs), and appear to be useful in prevention of the production of hazardous antibodies. However, whether immune responses can be regulated by monomeric sialoside ligands has not yet been known.

CD22 (also known as Siglec-2), a member of Siglec family, specifically recognizes α2,6 sialic acid, and is preferentially expressed in B lymphocytes (B cells) (24, 25). CD22 contains immunoreceptor tyrosine-based inhibition motifs (ITIMs) in the cytoplasmic region and negatively regulates signaling through BCR by recruiting SH2 domain-containing phosphatases at the phosphorylated ITIMs. CD22 expressed on B cells are mostly bound by α2,6 sialic acids expressed on the same cell (cis-ligand) (26), but can interact with exogenous α2,6 sialic acids (trans-ligand). Interaction with trans-ligands on antigen-expressing cells enhances CD22-mediated signal inhibition by co-ligating BCR and CD22 (27, 28). Interaction with endogenous ligands, most likely cis-ligands, is also suggested to regulate the signaling function of CD22. Early studies with B cell lines suggested that the ligand augments signal inhibition mediated by CD22 (3, 29). However, B cells that lack expression of α2,6 sialic acid due to targeted mutation of ST6GalI, a sialyl transferase required for synthesis of α2,6 sialic acid, showed reduced BCR signaling due to augmented CD22-mediated signal inhibition (30, 31), suggesting that the endogenous ligand down-modulates the signal inhibition activity of CD22. This conclusion was further supported by the finding that BCR signaling is reduced in B cells expressing CD22 that lacks ligand-binding capacity (32, 33).

Previously, we developed the C2/C9-modified synthetic sialoside GSC718 [compound 8 in Ref. (34)] that binds to both human and mouse recombinant CD22 with a 10,000 times higher affinity (100 nM) than the natural ligand α2,6 sialic acid. GSC718 binds to CD22 on the cell surface as well because GSC718 inhibits proximity labeling of CD22 ligands using B cells (35). To address whether a monomeric synthetic high-affinity sialoside ligand of CD22 can regulate immune responses, we synthesized fluorine-substituted form of GSC718 (GSC839) because fluorine affects various aspects of compounds including metabolism and binding affinity (36), and examined the effect of GSC718 and GSC839 on immune responses *in vitro* and that of GSC839 *in vivo*. These synthetic sialosides inhibited BCR ligation-induced B cell activation by reversing ligand-mediated regulation of CD22. By contrast, both GSC718 and GSC839 enhanced B cell activation induced by TLR ligands and antibody production by ligand-independent mechanism. Thus, CD22-binding synthetic sialosides regulate CD22 either positively or negatively, and by ligand-dependent and independent mechanisms depending on the stimuli, thereby regulating B cell activation and antibody responses *in vivo*. Interestingly, homology modeling of CD22 complexed with GSC839 suggests interactions between aromatic moieties of GSC839 with aromatic amino acid residues in CD22 account for high-affinity binding of GSC839 to CD22 in the absence of druggable pockets. Thus, our results suggest how to design chemical compounds that regulate members of the Siglec family.

## Materials and Methods

### Mice

C57BL/6 mice were purchased from Sankyo Labo Service Corporation, Inc. CD22^−/−^ (37) and ST6GalI^−/−^ (38) mice on a C57BL/6 background were described previously. Mice were used at 8–10 weeks old unless otherwise specified. All mice used in this study were bred and maintained in a specific pathogen-free animal facility of Tokyo Medical and Dental University. All procedures followed the guidelines of Tokyo Medical and Dental University for animal research and were approved by Institutional Animal Care and Use Committee, Tokyo Medical and Dental University.

### Synthetic Sialosides

Synthetic sialosides GSC718 were synthesized as previously described (6, 34). GSC839 was synthesized *via* 11 steps starting from the glycosylation of 4-fluorobenzyl alcohol with 5*N*-TFAc,9-*N*_3_-modified sialyl thioglycoside donor. The glycosylation afforded a mixture of α- and β-sialosides that were separated *via* 1,5-lactamization. Acetylation of the α-4-fluorobenzyl sialoside followed by selective removal of *N*-acetyl group with hydrazine acetate gave the lactamized compound as a single product in good yields. Subsequent introduction of the Boc group at the C5 position afforded the fully protected sialoside derivative. Lactam opening by treatment of NaOMe in anhydrous MeOH was achieved successfully to give the desired triol product in the ^2^*C*_5_ conformation in excellent yield. Next, the reduction of the azide functionality with triphenylphosphine in THF-H_2_O followed by the condensation with the carboxylic acid derivative produced the amide product in good yields over two steps. Finally, the conversion of the Boc carbamate into the acetoxyglycolyl amide at C5 position and subsequent global deprotection furnished the target GSC839 in good yields. Synthesis of GSC839 is described in more detail in Presentation S1 in Supplementary Material.

### Cell Isolation and Culture

Mouse spleen B cells were prepared as described previously (39). Peritoneal exudate cells were collected by intraperitoneal injection of ice-cold RPMI-1640 medium. In some experiments, peritoneal exudate cells were incubated in 24-well cell culture plate for 1 h. After discarding non-adherent cells, adherent cells were collected by treatment with Trypsin/EDTA (Nakalai). For preparation of total spleen cells, mouse spleen was incubated in Hank’s balanced salt solution (Wako) containing 0.1% collagenase (Sigma) at 37°C for 20 min, and minced. Cells were then collected. Bone marrow cells were obtained from mouse femur and tibia by flushing with RPMI-1640 medium. For preparation of bone marrow-derived DCs (BMDCs), 1 × 10^6^ bone marrow cells were cultured in 1 ml RPMI-1640 medium supplemented with 10% FCS (Nichirei Biosciences) 50 µM 2-mercaptoethanol (Sigma), and 1% penicillin/streptomycin (Nakalai) (complete RPMI-1640 medium) containing 200 ng/ml human Flt3L (Peprotech) or 10 ng/ml human GM-CSF (Peprotech) for 6–9 and 6 days, respectively. Total spleen cells and BMDCs were cultured with CpG oligo or LPS in the presence or absence of 80 µM GSC718 or GSC839 for 24 h.

### Cell Proliferation Assay

Cells were labeled with 10 µM carboxyfluorescein diacetate succinimidyl ester (CFSE) (molecular probes) for 10 min. CFSE-labeled or unlabeled cells (2 × 10^5^) were cultured in 200 µl complete RPMI-1640 medium in 96-well plate with CpG oligonucleotides (CpG oligo) (ODN1668) (Hokkaido System Science), F(ab′)_2_ fragments of goat anti-mouse IgM antibody (Jackson ImmunoResearch), LPS (Sigma, *E. coli*. O111:B4), or anti-CD40 antibody (FGK45) (40) (a kind gift of Dr. Rolink), in the presence or absence of 50 µM GSC718 or GSC839 for 72 h. Percentages of cells with reduced CFSE fluorescence were measured as divided cells.

### Assay for B Cell Proliferation That Depends on Both BCR and CD40 Ligation

Mouse spleen B cells were labeled with CFSE. Cells were cultured in complete RPMI medium with or without 1, 3, or 10 µg/ml anti-CD40 antibody (FGK45) and 10 μg/ml F(ab′)_2_ fragments of goat anti-mouse IgM antibody (Jackson ImmunoResearch). After 5 h, cells were washed twice and then cultured with or without 1, 3, and 10 µg/ml anti-CD40 antibody (FGK45). Cells were cultured in total 72 h and analyzed by flow cytometry.

### Flow Cytometry

Cells were incubated with anti-FcγRII/III antibody 2.4G2 for 10 min to block FcγRII/III-mediated binding, and stained for 30 min with the following antibodies: FITC-conjugated anti-mouse B7.2 (GL1, BD Pharmingen), biotin-conjugated MHC class II I-A/I-E (2G9, BD Pharmingen), PE-labeled streptavidin (BioLegend), Alexa647-conjugated anti-mouse F4/80 (BM8, BioLegend), eFluor450-conjugated anti-mouse CD11c (N418, eBioscience), biotinylated anti-mouse CD22 (F239), biotinylated anti-mouse Gr-1 (RB6-8C5), Pacific blue-conjugated anti-mouse CD45R (RA3-6B2), Alexa647- or FITC-conjugated anti-mouse CD3 (145-2C11, BioLegend), FITC-conjugated anti-mouse CD11b (M1/70, BioLegend), and FITC-conjugated anti-mouse CD19 (eBio1D3, eBioscience). Cells were washed twice with PBS containing 2% FCS (FACS buffer) and then suspended in FACS buffer. All these procedures were done on ice. In peritoneal cells, total macrophages (CD11b^+i^ F4/80^+i^), large peritoneal macrophages (LPM) (CD11b^hi^ F4/80^hi^), small peritoneal macrophages (SPM) (CD11b^+^ F4/80^lo^), and neutrophils (CD11b^+^ F4/80^−^, Gr-1^+^) were defined. CD11b^+^Gr1^+^ bone marrow cells were gated as bone marrow granulocytes. Spleen dendritic cells (DCs) were defined as CD3^−^CD19^−^NK1.1^−^CD11c^+^. Spleen T and B cells were defined as CD3^+^B220^−^ and CD3^−^B220^+^ cells in lymphocyte gate (FSC^lo^, SSC^lo^). Cells were analyzed using a CyAn (Beckman Courter) or FACSVerse™ (BD).

### Competition ELISA for Binding of Sialosides to CD22

Recombinant proteins composed of the amino-terminal domains (domains 1–3) of mouse or human CD22 and the Fc region of human IgG1 (CD22-Fc) were described previously (41). Microtiter plates (96 well) were coated with 20 µg/ml α1-acid glycoprotein (Sigma). Alternatively, plates were coated with 50 µg/ml streptavidin followed by incubation with 4 µg/ml biotinylated synthetic CD22 ligand (42). Plates coated with α1-acid glycoprotein and synthetic CD22 ligand were then blocked with PBS containing 1% bovine serum albumin, followed by incubation with human or mouse CD22-Fc and compounds for 2 h, respectively. CD22-Fc bound to the plates was detected using alkaline phosphatase (AP)-conjugated goat anti-human IgG (Southern Biotechnology) and AP substrate solution (Sigma). The optical density at 405 nm was measured by a microplate reader (Molecular Devices). The concentrations of the compounds that reduce the binding of CD22-Fc to the biotinylated CD22 ligand and α1-acid glycoprotein by 50% (IC50) were determined.

### Measurement of TNFα

TNFα and IL-6 were measured by TNFα ELISA kit and IL-6 ELISA kit (BioLegend), respectively, according to the manufacture’s protocol.

### Immunization

Mice were intraperitoneally or subcutaneously immunized with 2.5 or 10 µg ovalbumin (OVA) (Sigma) in PBS with or without 100 µg GSC839, 20, 50, or 100 µg CpG oligo or 0.15, 0.5, or 1.5 µl Alhydrogel (In vivoGen) containing 3, 10, or 30 µg alum, respectively. Blood samples were collected after 24 h for measurement of cytokine production and after 3 weeks for measurement of OVA-specific antibody production. In some mice, 2.5 µg OVA was injected subcutaneously without any adjuvants at 9 weeks after primary immunization to induce memory responses (43). Serum samples were collected 1 week later for measurement of OVA-specific antibodies.

### Measurement of Anti-OVA IgG

Microtiter plates (96 well) were coated with 100 µg/ml OVA at 4°C overnight. Plates were washed with PBS twice, and blocked with PBS containing 1% bovine serum albumin for 2 h at room temperature. After discarding the blocking buffer, serially diluted serum samples were added to the wells, and incubated for 2 h at room temperature. After washing with PBS containing 0.05% tween 20 (PBS-T) five times, wells were incubated with alkaline phosphatase (AP)-conjugated goat anti-mouse IgG, IgG1, IgG2b (Southern Biotechnology) for 1 h at room temperature. Alternatively, wells were incubated with biotin-conjugated goat anti-mouse IgG2c (Abcam) for 1 h, followed by incubation with AP-conjugated streptavidin for 30 min. After washing with PBS-T five times, wells were incubated with AP substrate solution (Sigma). After incubation at room temperature for approximately 20 min, optical density at 405 nm was measured by microplate reader (Molecular Devices).

### Statistical Analysis

Data of *in vitro* experiments were analyzed by unpaired two-tailed *t*-test. Data of *in vivo* immunization were analyzed by Mann–Whitney test, Wilcoxon signed-rank test, or Kruskal–Wallis test. All the analysis was done using GraphPad PRISM software (GraphPad) or EZR. *P*-values <0.05 were regarded as statistically significant.

### Homology Modeling

A homology model of CD22 were generated by SWISS-MODEL Workspace (44) using the coordinates of the ligand-binding domain of human CD22 (45) (PDB ID 5VKM, residues 20–138) as a template structure. The figures of the sequence alignment and molecular models are prepared using ESPript (46) and PyMOL (The PyMOL Molecular Graphics System, Schrödinger, LLC.), respectively.

## Results

### CD22-Binding Sialosides GSC718 and GSC839 Enhance B Cell Proliferation Induced by TLR Ligands or CD40 Ligation, but Downregulate BCR Ligation-Induced B Cell Proliferation

**Figure 1:**
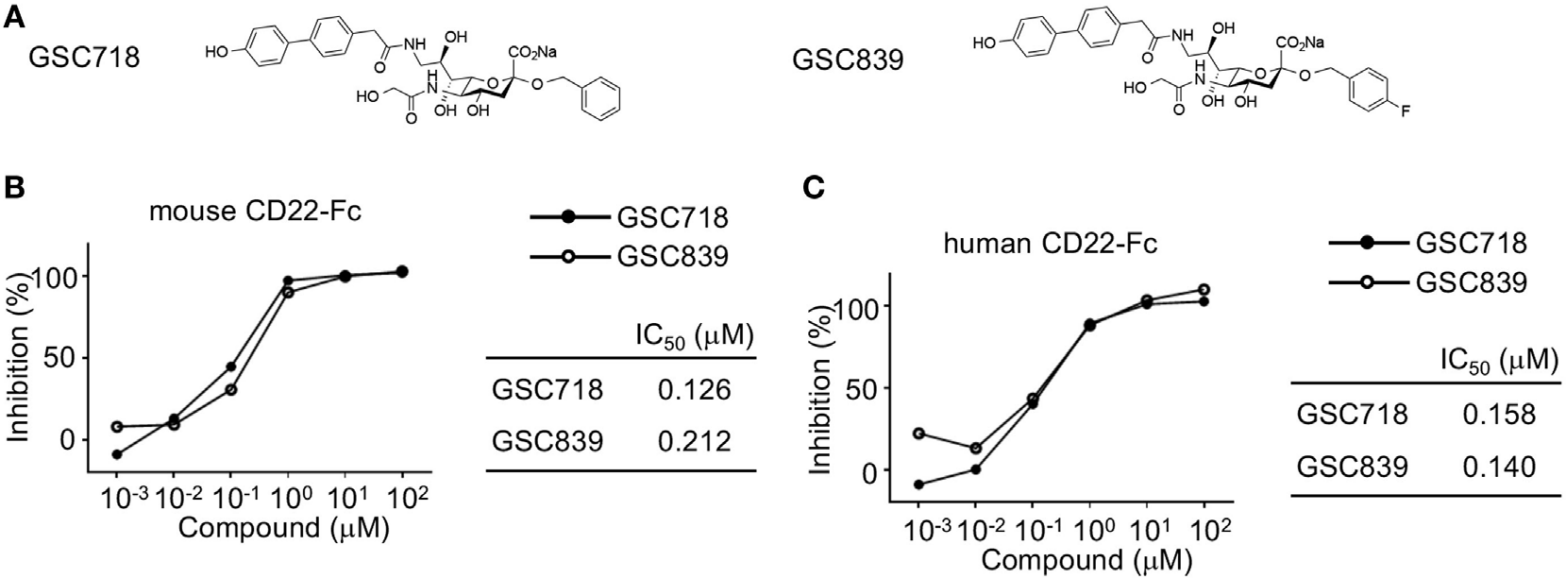
High-affinity binding of GSC718 and GSC839 to CD22. **(A)** Structure of GSC718 and GSC839. **(B,C)** Competition ELISA for measuring affinity of GSC718 and GSC839 to ligand. Indicated concentrations of GSC718 (closed circles) or GSC839 (open circles) and mouse **(B)** or human CD22-Fc **(C)** protein were incubated in wells coated with synthetic CD22 ligand **(B)** or α1-acid glycoprotein **(C)**, and binding of CD22-Fc to these ligands was detected by ELISA. Percent binding inhibition by the sialosides, and the concentration of sialosides that achieves 50% binding inhibition (IC_50_) were calculated.

We synthesized the novel sialoside GSC839 by introducing fluorine atom into GSC718 at the C-4 position of the benzyl group (Figure 1A). When we measured the binding affinity of GSC839 to CD22 by a competition ELISA using the fusion protein containing the ligand-binding domain of CD22 and the Fc portion of human IgG (CD22-Fc), GSC839 bound to both human and mouse CD22 with comparable affinities (IC_50_ ~100 nM) and these affinities are similar to those of GSC718 (Figures 1B,C).

**Figure 2:**
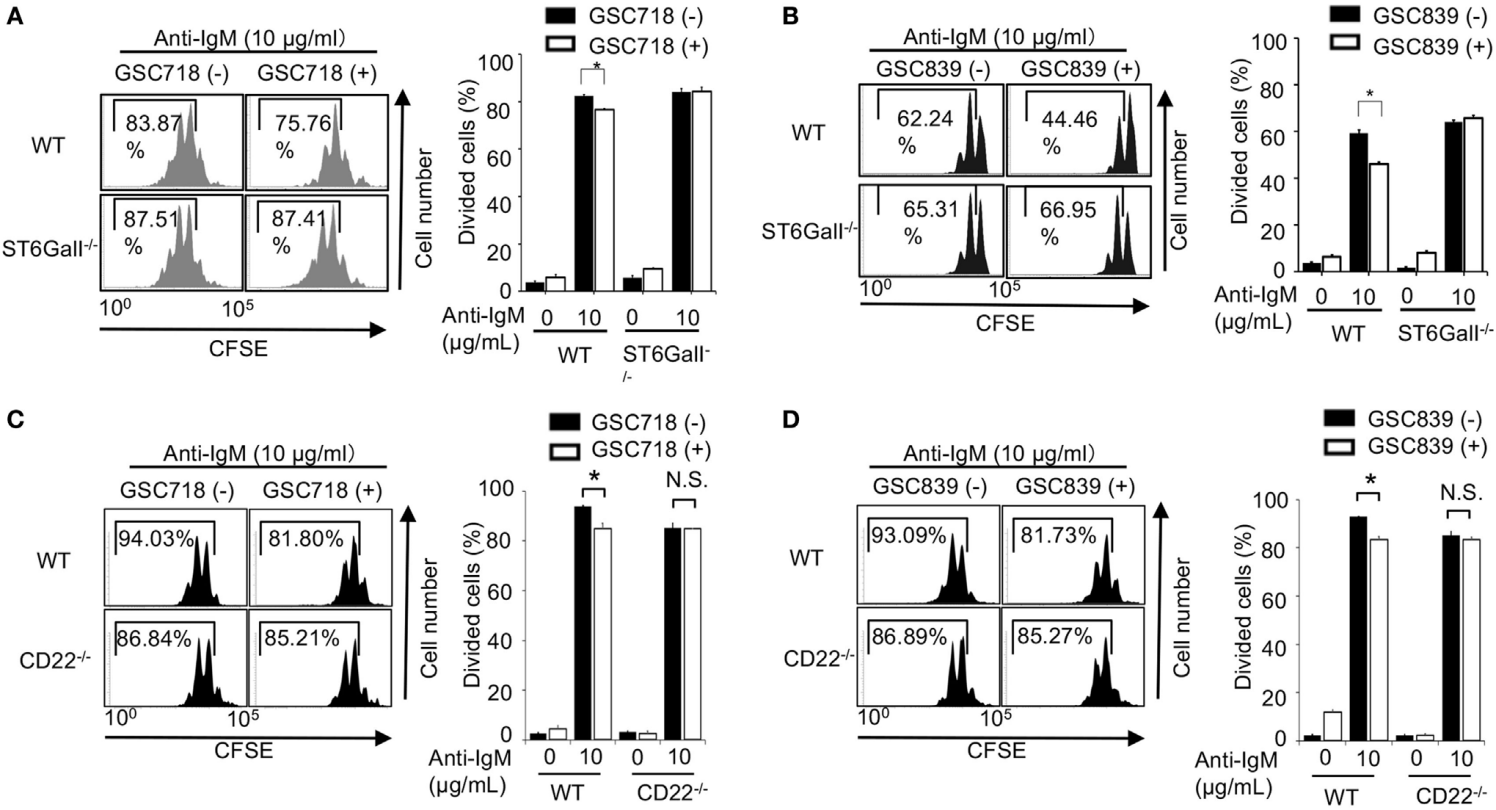
GSC718 and GSC839 downregulate B cell proliferation induced by BCR ligation *in vitro*. Spleen B cells obtained from wild type (WT) **(A–D)**, CD22^−/−^ **(C,D)**, and ST6GalI^−/−^ **(A,B)** C57BL/6 mice were stained with carboxyfluorescein diacetate succinimidyl ester (CFSE) and cultured for 72 h in the presence of indicated concentrations of anti-IgM with or without 50 µM GSC718 **(A,C)** or GSC839 **(B,D)**. Cells were analyzed by FCM and percentages of proliferated cells are indicated (left panels). Data are representative of at least three experiments. Mean ± SD (*n* = 3) is shown (right panels). Data were analyzed by unpaired *t*-test. *P < 0.05, NS, not significant.

We addressed whether the CD22-binding sialosides modulate B cell proliferation. We first addressed the effect of the CD22-binding sialosides on BCR ligation-induced B cell proliferation. Treatment with GSC718 or GSC839 modestly but significantly reduced proliferation of wild-type (WT) B cells stimulated with anti-IgM (Figures 2A–D). Although ST6GalI^−/−^ B cells were previously shown to be activated less efficiently by various stimuli (31, 38), anti-IgM induced proliferation of ST6GalI^−/−^ B cells as efficiently as WT B cells probably because of a different assay to address cell proliferation from the previous studies. Treatment with GSC718 or GSC839 did not alter proliferation of anti-IgM-stimulated ST6GalI^−/−^ B cells (Figures 2A,B) or CD22^−/−^ B cells (Figures 2C,D), suggesting that the effect of GSC718 and GSC839 to reduce BCR ligation-induced B cell proliferation requires both CD22 and CD22 ligands. Thus, CD22-binding sialosides down-modulate BCR ligation-induced B cell proliferation by reversing ligand-mediated regulation on CD22.

**Figure 3:**
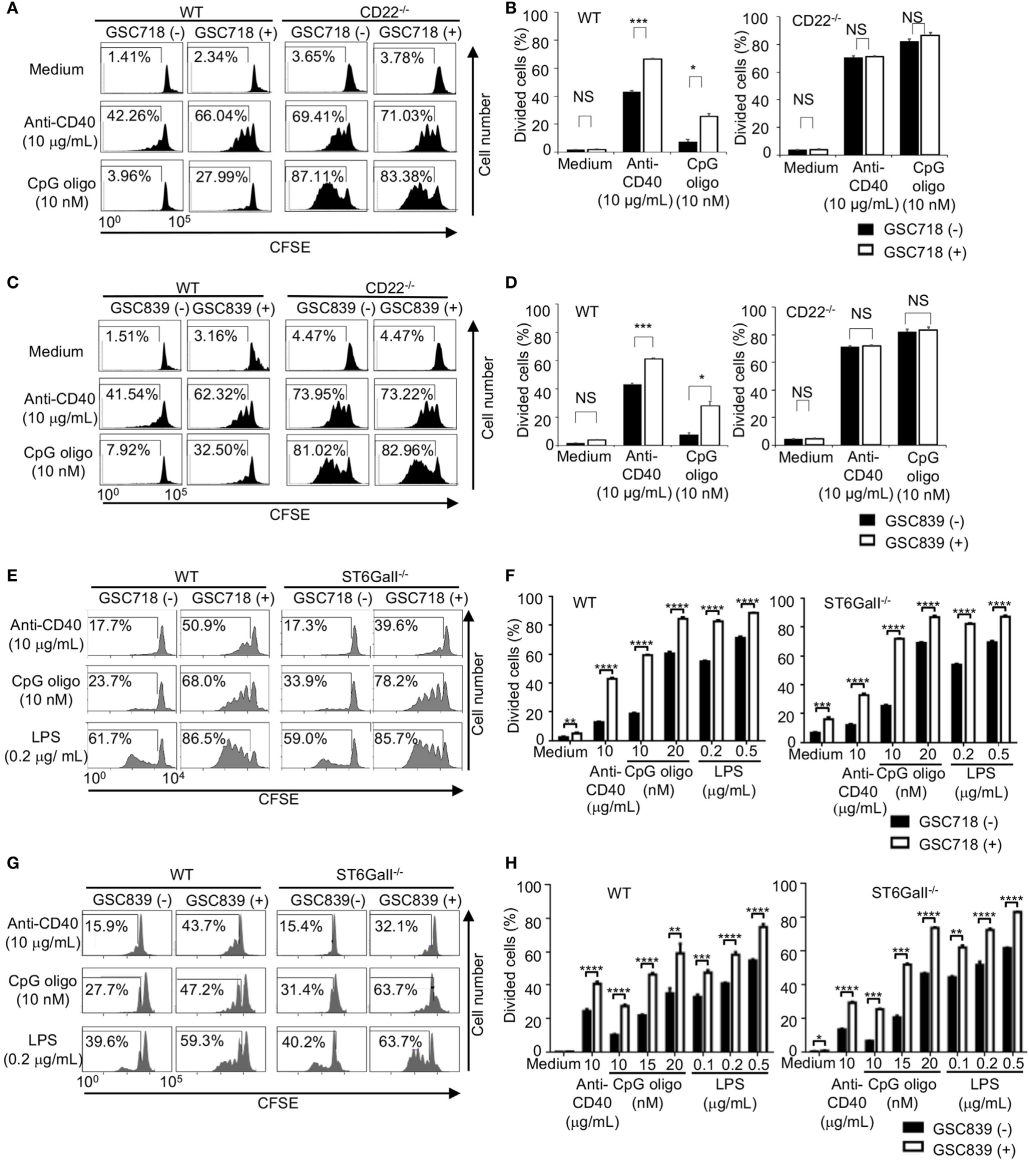
GSC718 and GSC839 augment B cell proliferation induced by anti-CD40 and TLR ligands *in vitro*. Spleen B cells obtained from wild type (WT) **(A–H)**, CD22^−/−^ **(A–D)**, and ST6GalI^−/−^ **(E–H)** C57BL/6 mice were stained with carboxyfluorescein diacetate succinimidyl ester (CFSE) and cultured for 72 h in the presence of indicated concentrations of anti-CD40 **(A–H)**, CpG oligo **(A–H)**, or LPS **(E–H)** with or without 50 µM GSC718 **(A,B,E,F)** or GSC839 **(C,D,G,H)**. Cells were analyzed by FCM and percentages of proliferated cells are indicated **(A,C,E,G)**. Data are representative of at least three experiments. Mean ± SD (*n* = 3) is shown **(B,D,F,H)**. Data were analyzed by unpaired *t*-test. **P < 0.01, ***P < 0.001, NS, not significant.

Because CD22^−/−^ B cells show an augmented response to TLR ligands and CD40 ligation (33, 47–49), we examined whether GSC718 and GSC839 modulate activation of B cells treated with agonistic anti-CD40 antibody or the TLR ligands CpG oligo and LPS. Upon stimulation with anti-CD40 antibody or CpG oligo, CD22^−/−^ B cells showed augmented proliferation compared to WT B cells in agreement with the previous findings (33, 47, 48) (Figures 3A–D). Treatment with GSC718 or GSC839 enhanced proliferation of WT B cells stimulated with anti-CD40 antibody or low-dose CpG oligo probably by inhibiting CD22-mediated signal inhibition. By contrast, both GSC718 and GSC839 failed to augment proliferation of CD22^−/−^ B cells, suggesting that these sialosides require CD22 for enhancing B cell activation. Thus, GSC718 and GSC839 enhance proliferation of B cells stimulated with CD40 ligation or CpG oligo by reversing CD22-mediated inhibition of B cell activation.

To address the role of endogenous CD22 ligands in the B cell responses to CD40 ligation and TLR ligands, we examined the responses of ST6GalI^−/−^ B cells. When stimulated with anti-CD40 antibody or low-dose TLR ligands, such as LPS and CpG oligo, proliferation of ST6GalI^−/−^ B cells was comparable to that of WT B cells (Figures 3E–H).This result indicates that CD22 ligands do not regulate B cell proliferation induced by CD40 ligation or TLR ligands. Treatment with GSC718 augmented proliferation of ST6GalI^−/−^ B cells induced by TLR ligands (Figures 3E,F) as strongly as that of WT B cells, indicating that GSC718 augments activation of TLR ligands-stimulated B cells by a ligand-independent pathway. GSC718 also augmented proliferation of anti-CD40-stimulated B cells. In the presence of anti-CD40 and GSC718, the proliferation of ST6GalI^−/−^ B cells was comparable to but slightly less than that of WT B cells. Essentially the same results were obtained with treatment with GSC839 (Figures 3G,H). Thus, CD22-binding sialosides appear to augment B cell proliferation by reversing inhibitory function of CD22 independently of CD22 ligands.

Taken together, both GSC718 and GSC839 augment B cell proliferation induced by TLR ligands or anti-CD40 in a manner independent on CD22 ligands, but down-modulates BCR ligation-induced B cell proliferation by reversing ligand-mediated regulation on CD22.

### GSC839 Augments B Cell Proliferation Induced by Combination of CD40 Ligation and BCR Ligation

**Figure 4:**
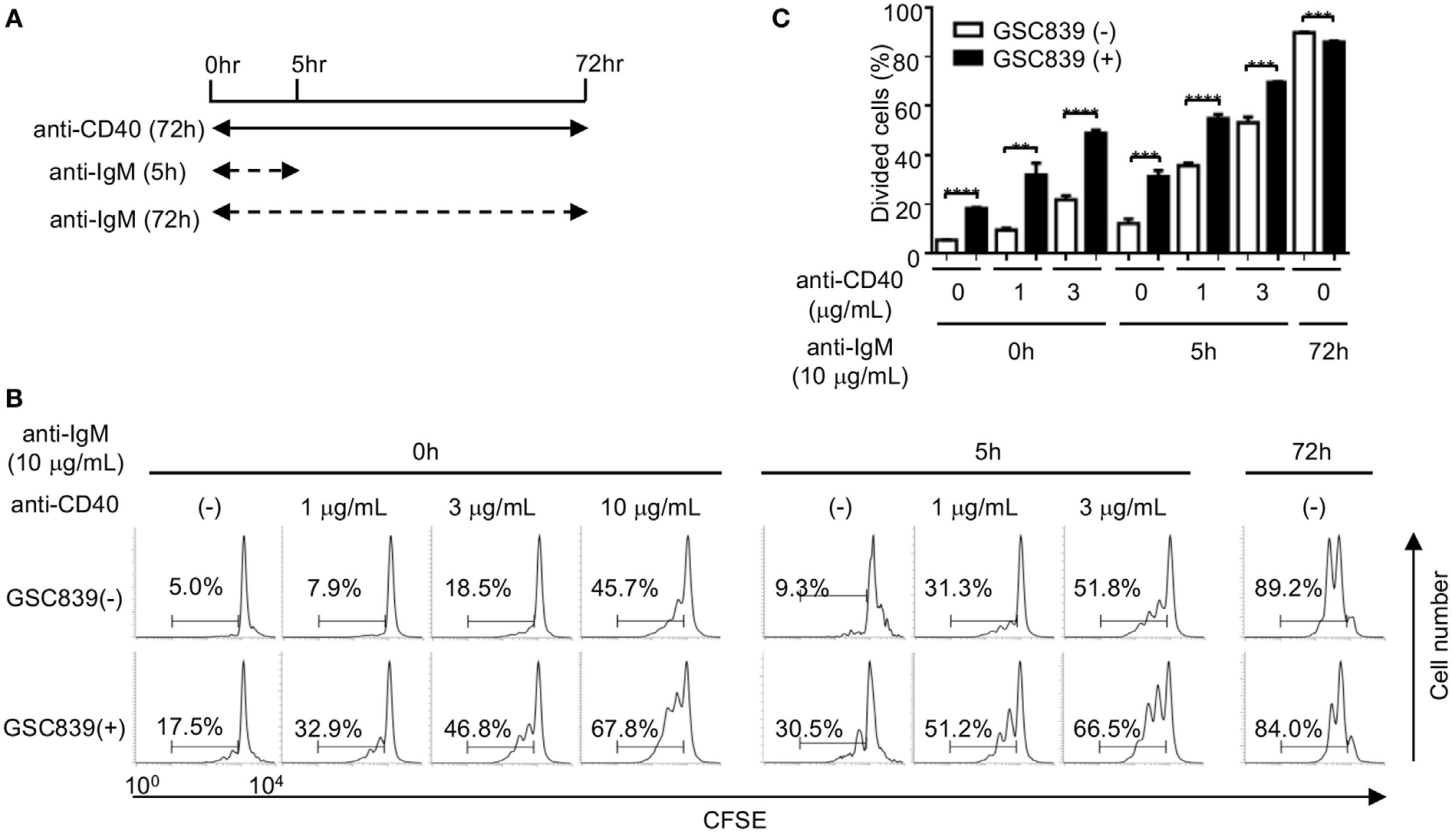
GSC839 augments proliferation of B cells stimulated with anti-IgM together with anti-CD40. Spleen B cells obtained from wild-type C57BL/6 mice were stimulated with 10 µg/ml anti-IgM for either 72 h or initial 5 h together with indicated concentrations of anti-CD40 for 72 h. Schematic diagram illustrating time course of B cell stimulation **(A)**. Cells were analyzed by FCM and percentages of proliferated cells are indicated **(B)**. Data are representative of three experiments. Mean ± SD (*n* = 3) is shown **(C)**. Data were analyzed by unpaired *t*-test. **P < 0.01, ***P < 0.001.

When B cells respond to antigens, antigen-induced BCR signaling alone does not induce proliferation of B cells (50), and proliferation of antigen-stimulated B cells requires co-stimulatory signaling through CD40 (51). Continuous BCR ligation by anti-IgM antibody for 72 h induces B cell proliferation. However, treatment with anti-IgM antibody for the first 5 h of the culture does not induce B cell proliferation by itself, but induces B cell proliferation in the presence of a low dose anti-CD40 antibody (1 or 3 µg/ml), whereas the low dose anti-CD40 alone induces only marginal proliferation (Figures 4A–C). Thus, in the culture condition where B cells are stimulated with anti-IgM for 5 h together with a low-dose anti-CD40 for 72 h, B cell proliferation requires both BCR ligation and CD40 signaling as is the case for *in vivo* B cell responses to antigens. Activity of GSC839 in binding to CD22 and inducing B cell proliferation is similar to that of GSC718. Thus, we chose GSC839 simply due to availability for *in vivo* study and added GSC839 to this culture. B cell proliferation induced by treatment with anti-IgM antibody for the first 5 h together with the low-dose anti-CD40 was further enhanced by GSC839, suggesting that GSC839 enhances B cell activation that depends on both BCR ligation and CD40 signaling.

### GSC718 and GSC839 Do Not Activate Innate Immune Cells

**Figure 5:**
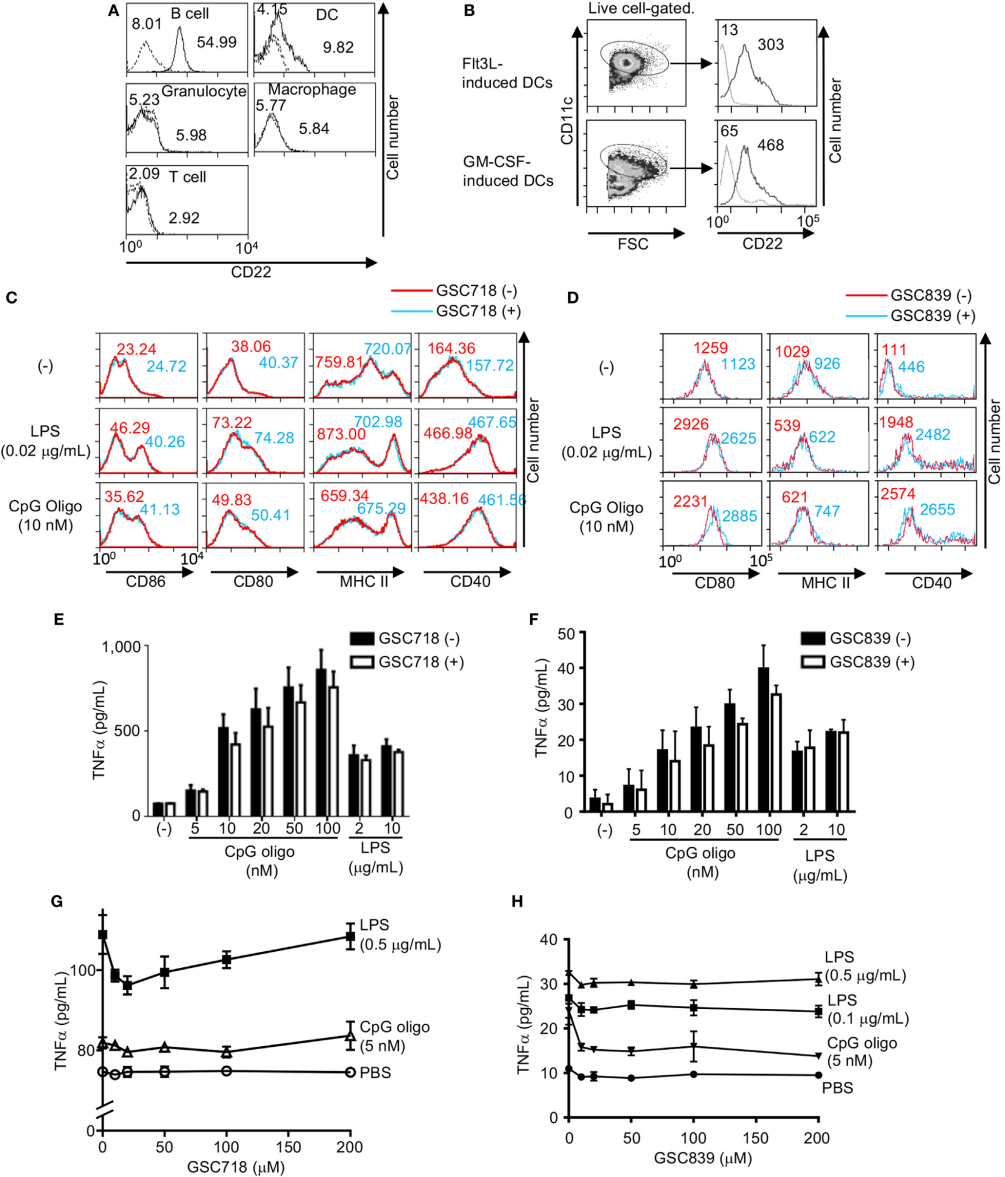
GSC718 does not enhance TNFα production. **(A)** CD22 expression in various cells. PEC, BM cells, and splenocytes were obtained from wild type (solid lines) or CD22^−/−^ C57BL/6 mice (dotted lines). F4/80^+^CD11c^+^ peritoneal macrophages, Gr1^+^ BM granulocytes, CD3^−^CD19^−^NK1.1^−^CD11c^+^ spleen dendritic cells (DCs), CD3^−^B220^+^ spleen B cells, CD3^+^B220^−^ spleen T cells were analyzed for CD22 expression by FCM. MFIs are indicated. **(B–D)** GSC718 does not activate DCs. DCs were generated by culturing bone marrow cells from C57BL/6 mice with Flt3L or GM-CSF and were analyzed for CD22 and CD11c by FCM **(B)**. Gating strategy of CD11c^+^ cells and expression level of CD22 in comparison to FMO control (dotted line) in CD11c^+^ cells are shown. MFIs are indicated. Cells were stimulated with 10 nM CpG oligo or 0.02 µg/ml LPS with (red) or without (blue) 80 µM GSC718 **(C)** or GSC839 **(D)**, and expression of CD86 **(C)**, CD80, MHCII, and CD40 **(C,D)** was analyzed by FCM. MFI is indicated. Representative data of three experiments. **(E–H)** TNFα production *in vitro*. Whole spleen cells were obtained from C57BL/6 mouse and cultured for 24 h with indicated concentrations of CpG or LPS in the presence (gray columns) or absence (white columns) of 50 µM GSC718 **(E)** or GSC839 **(F)**, or indicated concentrations of GSC718 **(G)** or GSC839 **(H)**. *n* = 3. Error bars show mean ± SD.

To address whether CD22-binding sialosides activate other immune cells than B cells, we examined expression of CD22 in various immune cell types. Splenic DCs expressed CD22 at a level lower than B cells do in agreement with the previous finding (52), whereas CD22 was not expressed in the other innate immune cells such as macrophages and granulocytes (Figure 5A). To further address CD22 expression on DCs, we generated DCs by culturing mouse bone marrow cells with Flt3L or GM-CSF. CD22 expression was detected in both Flt3L- and GM-CSF-induced DCs (Figure 5B).

To address whether GSC718 and GSC839 activate DCs, we stimulated GM-CSF-induced BM DCs with GSC718 in the presence or absence of small amounts of TLR ligands, and examined expression of activation markers such as CD80, CD86, CD40, and MHCII because GM-CSF-induced BM DCs showed higher CD22 expression than spleen DCs. Although treatment with TLR ligands alone enhanced expression of CD80, CD86, and CD40, almost no upregulation of these markers were induced by GSC718 and GSC839 regardless of presence or absence of TLR ligands (Figures 5C,D). MHCII was not augmented by the treatment with TLR ligands at the tested concentrations. Thus, CD22-binding sialosides fail to activate DCs probably because of low CD22 expression.

Next, we addressed whether these CD22-binding sialosides induce inflammatory responses. When we cultured total mouse spleen cells with GSC718 or GSC839, the level of TNFα was not increased in culture supernatant whereas TLR ligands such as CpG oligo and LPS induced TNFα production in these cells (Figures 5E–H). As GSC718 and GSC839 enhance activation of B cells induced by TLR ligands, we examined whether these sialosides enhance production of inflammatory cytokines induced by TLR ligands. However, neither GSC718 nor GSC839 enhanced TNFα production from total spleen cells stimulated with CpG oligo or LPS. Thus, CD22-binding sialosides do not induce inflammatory response by itself or enhance the inflammatory response to TLR ligands.

### GSC839 Carries an Adjuvant Activity

**Figure 6:**
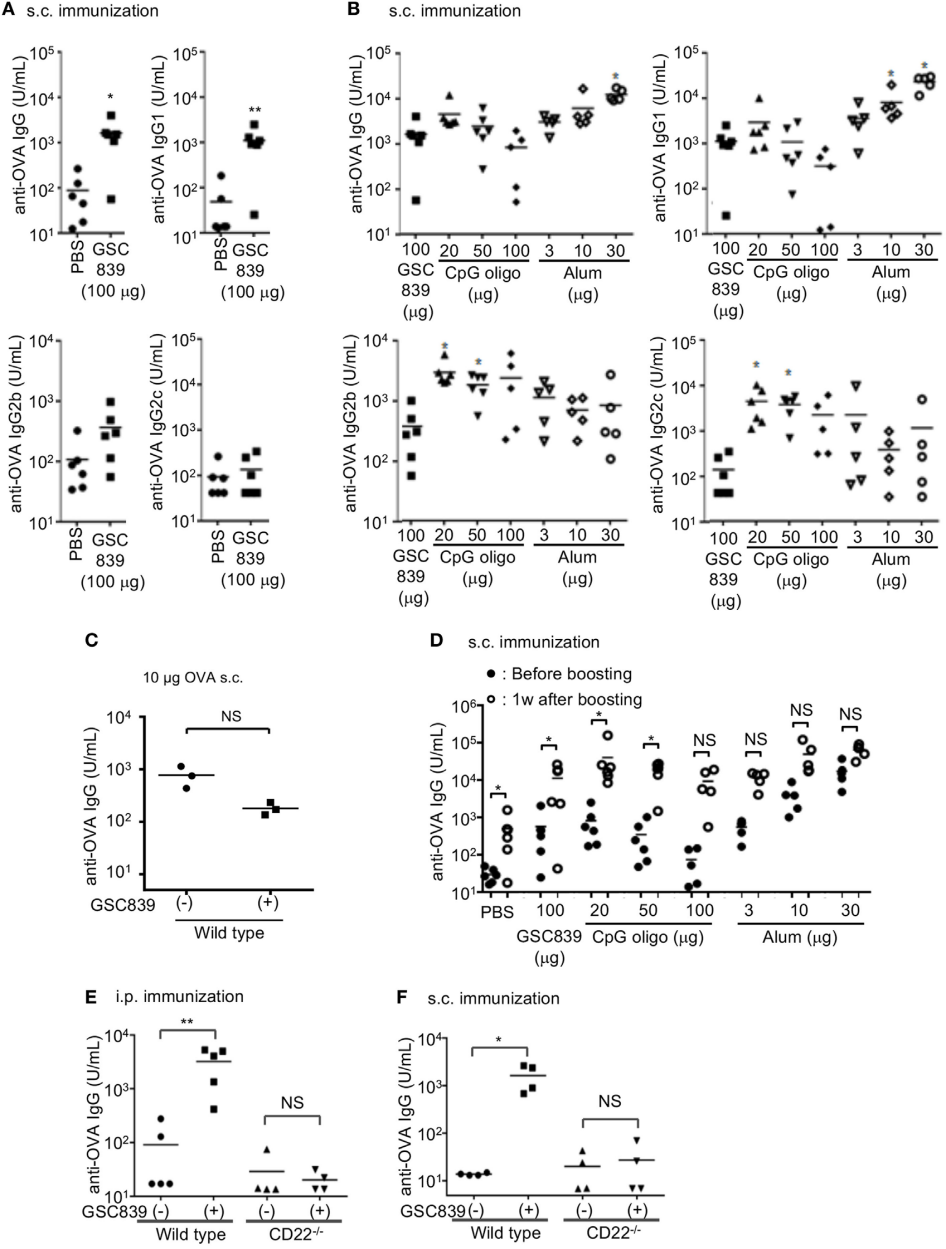
GSC839 promotes antibody production *in vivo*. **(A)** Augmented antibody production by GSC839. C57BL/6 mice were subcutaneously immunized with 2.5 µg ovalbumin (OVA) together with or without 100 µg GSC839. The titers of serum anti-OVA IgG, IgG1, IgG2b, and IgG2c at day 21 were measured by ELISA. Data were analyzed by Mann–Whitney test. *P < 0.05, **P < 0.01. **(B)** Comparison of the effect of GSC839 on antibody production with that of other adjuvants. C57BL/6 mice were subcutaneously immunized with 2.5 µg OVA together with the indicated amounts of GSC839, CpG oligo, or alum. The titers of serum anti-OVA IgG, IgG1, IgG2b, and IgG2c at day 21 were measured by ELISA. Difference between the antibody titers induced by GSC839 and those induced by LPS or CpG oligos was analyzed by Kruskal–Wallis test, and Steel test was applied as *post hoc* analysis. *P < 0.05. **(C)** GSC839 fails to augment antibody responses to a higher amount of antigen. C57BL/6 mice were subcutaneously immunized with 10 µg OVA together with or without 100 µg GSC839. The titers of serum anti-OVA IgG at day 21 were measured by ELISA. Data were analyzed by Mann–Whitney test. NS, not significant. **(D)** GSC839 augments immunological memory. C57BL/6 mice were subcutaneously immunized with 2.5 µg OVA together with indicated amounts of GSC839, CpG oligo, or alum. After 9 weeks, mice were intravenously boosted with 2.5 µg OVA. The titers of serum anti-OVA IgG before and 7 days after boost were measured by ELISA. Data were analyzed by Wilcoxon signed-rank test. *P < 0.05, **P < 0.01, NS, not significant. **(E,F)** CD22 is required for the adjuvant effect of GSC839. Wild type and CD22^−/−^ C57BL/6 mice at 7–11 weeks old were intraperitoneally **(E)** or subcutaneously **(F)** immunized with 2.5 µg OVA with or without 100 µg GSC839, and the titers of serum anti-OVA IgG at day 21 were measured by ELISA. Data were analyzed by Mann–Whitney test. *P < 0.05, **P < 0.01, NS, not significant.

Because GSC839 enhances *in vitro* B cell activation that depends on both BCR and CD40 signaling, we hypothesized that GSC839 enhances *in vivo* B cell responses to antigens as well. To address this possibility, we subcutaneously immunized mice with OVA together with GSC839 or known adjuvants such as CpG oligo and alum. Mice immunized with OVA together with GSC839 showed significantly higher antibody titers than those immunized with OVA alone (Figure 6A). The total anti-OVA IgG titers induced by GSC839 were comparable to those induced by CpG oligo and a small amount of alum, but lower than those induced by larger amounts of alum (Figure 6B). GSC-839 failed to enhance antibody production when mice were immunized with a higher amount of OVA (Figure 6C).

As IgG2b and IgG2c are more pro-inflammatory than IgG1, we addressed IgG subtypes of specific antibodies induced by GSC839. The titers of anti-OVA IgG1 induced by OVA together with GSC839 were significantly higher than those induced by OVA alone (Figure 6A), and comparable to those induced by OVA together with CpG oligo or a small amount of alum (Figure 5B). By contrast, GSC839 did not augment production of IgG2b or IgG2c (Figure 6A), and the titers of anti-OVA IgG2b and IgG2c induced by GSC839 were much lower than those induced by CpG oligo (Figure 6B).

We next addressed whether GSC839 augments immunological memory. We immunized mice with OVA together with GSC839, CpG oligo, or alum, and, after 9 weeks, boosted the mice with OVA alone. The antibody titer was significantly increased after boost compared to that before the boost in mice primed with OVA together with GSC839 as well as mice primed with OVA together with CpG oligo or a small amount of alum (Figure 6D). This result indicates that GSC839 augments immunological memory as well as primary antibody responses.

To address whether GSC839 augments antibody production by regulating CD22, we immunized mice with OVA together with GSC839 either intraperitoneally or subcutaneously. GSC839 augmented antibody production in WT but not CD22^−/−^ mice regardless of the route of immunization (Figures 6E,F). This result clearly indicates that GSC839 augments antibody production by regulating CD22.

### GSC839 Does Not Induce Inflammation in Mice

**Figure 7:**
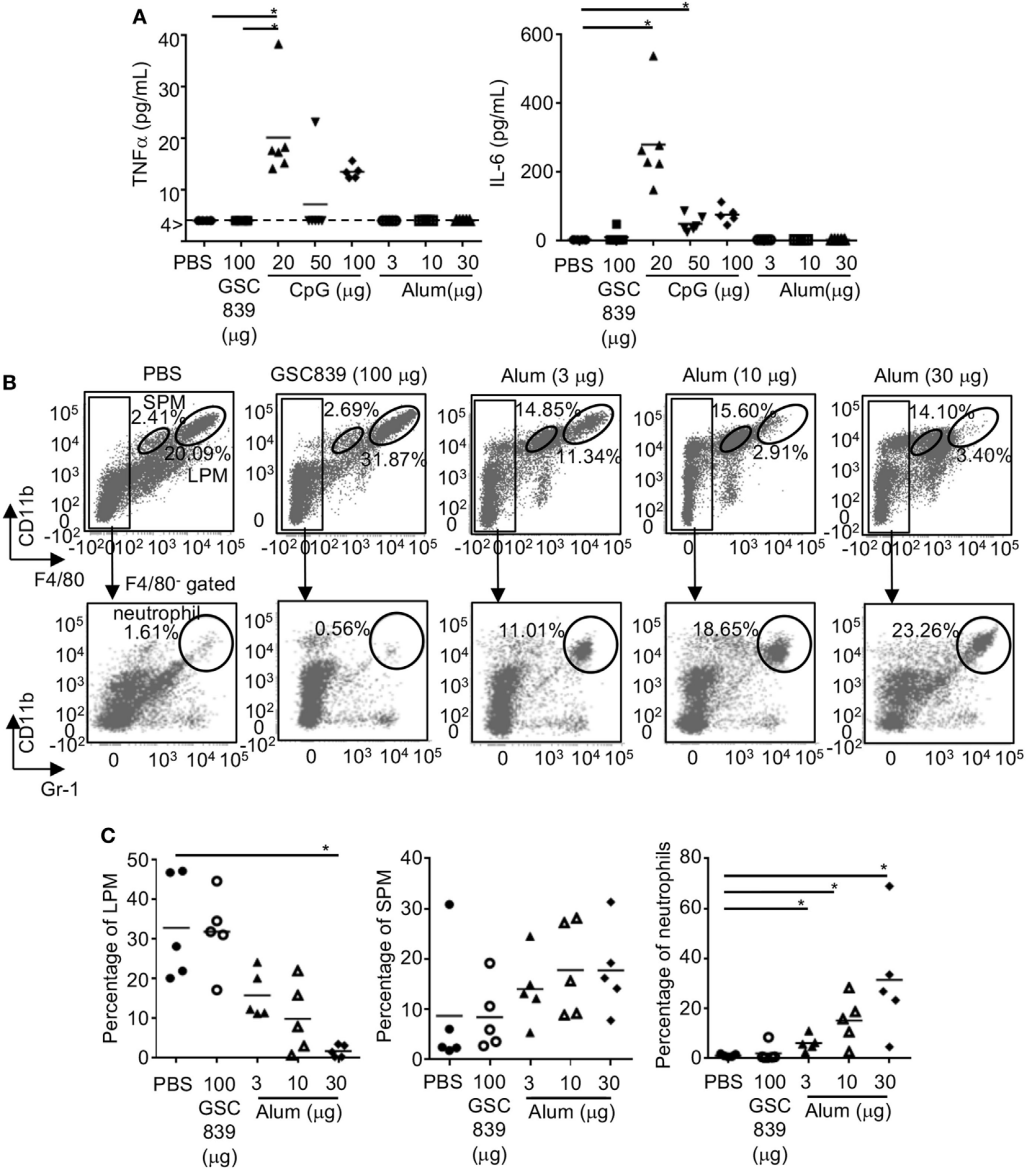
*In vivo* treatment with GSC839 does not induce inflammation. **(A)** Production of inflammatory cytokines. C57BL/6 mice were subcutaneously immunized with 2.5 µg ovalbumin together with indicated amounts of GSC839, CpG oligo, or alum. The levels of serum TNFα and IL-6 24 h after immunization were measured by ELISA. Data were analyzed by Kruskal–Wallis test and Steel analysis was applied as *post hoc* analysis. *P < 0.05. **(B,C)** C57BL/6 mice were intraperitoneally injected with indicated amounts of alum or GSC839. After 24 h, peritoneal cells were collected and analyzed for CD11b, F4/80, and Gr-1 by FCM. **(B)** Representative data. The percentages of large peritoneal macrophages (LPM) (CD11b^hi^ F4/80^hi^), small peritoneal macrophages (SPM) (CD11b^+^ F4/80^lo^), and neutrophils (CD11b^+^ F4/80^−^, Gr-1^+^) are indicated. **(C)** Combined data from five experiments. Percentages of indicated cells in total peritoneal cells are shown. Data were analyzed by Kruskal–Wallis test and Steel analysis was applied as *post hoc* analysis. *P < 0.05.

To address whether GSC839 induces inflammation *in vivo*, we subcutaneously immunized mice with OVA together with GSC839, CpG oligo, or alum, and measured cytokine production in sera. Both TNFα and IL-6 were produced by CpG oligo but not GSC839 or alum (Figure 7A). Next, we injected GSC839 and alum intraperitoneally, and analyzed peritoneal exudate cells. In peritoneal cells from mice treated with alum, the percentage of LPM (53), which are resident macrophages, was significantly reduced whereas the percentages of both SPM, which are blood-derive inflammatory macrophages, and neutrophils (53), were increased, suggesting that alum induces peritoneal inflammation (Figures 7B,C). By contrast, treatment with GSC839 did not alter the percentage of macrophages or neutrophils, clearly demonstrating that GSC839 does not induce inflammation *in vivo*.

### Homology Model of CD22 Bound by GSC839

**Figure 8:**
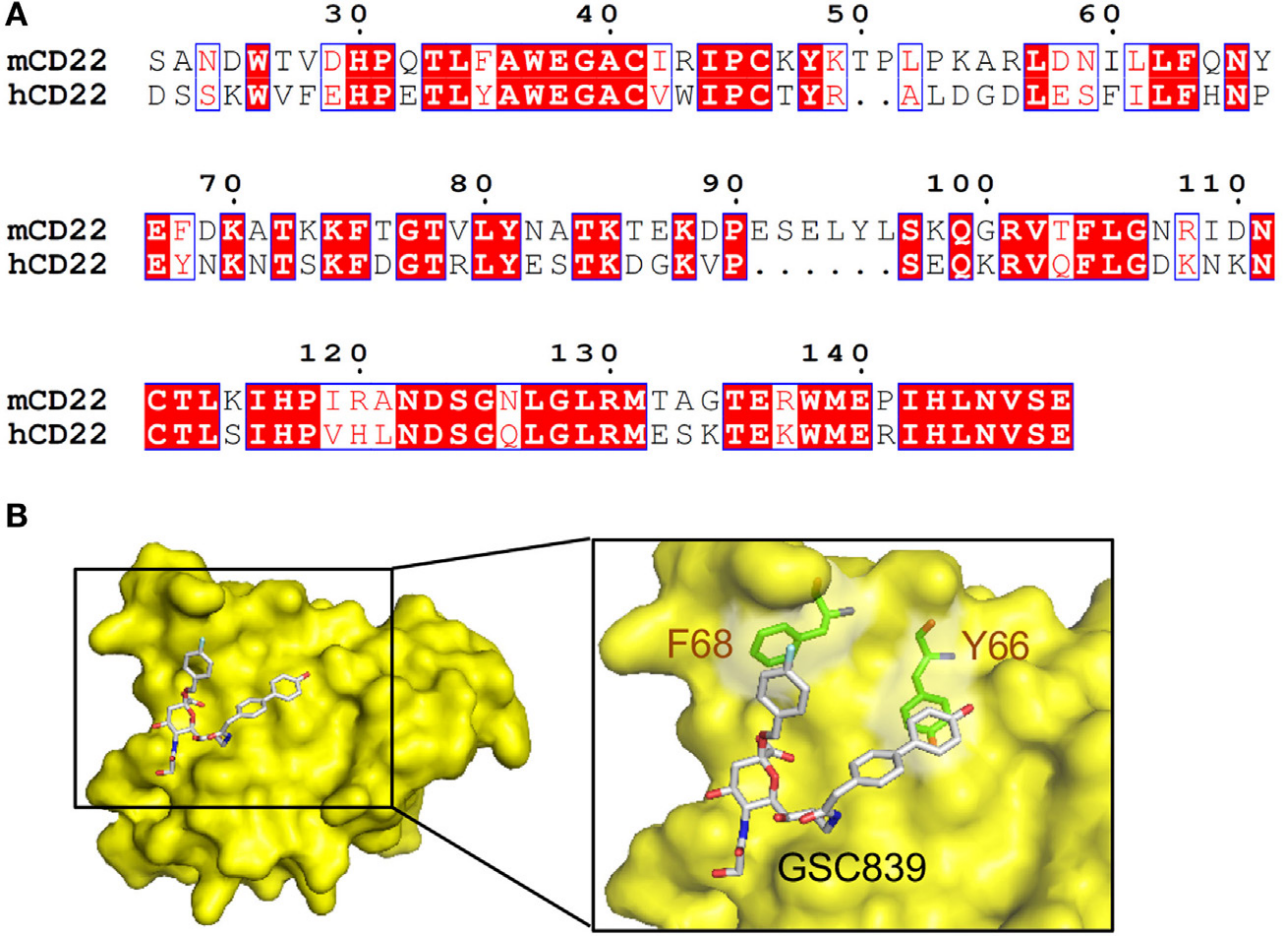
Homology modeling of CD22 complexed with GSC839. **(A)** Amino acid sequence alignment of the ligand-binding domain of mouse CD22 and human CD22. White characters on a red background are identical residues. Similar residues are highlighted as red characters and framed in blue. Sequence number of mouse CD22 are indicated every 10 residues. **(B)** Homology model of CD22 (surface model, yellow). Model of bound GSC839 is represented as a stick model. Aromatic residues that interact with GSC839 are indicated as green stick models.

We generated a homology model of the ligand-binding domain of CD22 based on the crystal structure of human CD22 (45). Amino acid sequence alignment between mouse and human CD22 (Figure 8A) show 53% identity. The overall structure of the obtained model of mouse CD22 (Figure 8B) is quite similar to that of human CD22 (45). We analyzed presence of potential binding pockets by MetaPocket 2.0 server (54). No pockets are present around conserved Arg130, which forms a salt bridge with the carboxylate group of sialic acid.

Next, we generated the model of CD22 complexed with GSC839 (Figure 8B). We used structures of Me-α-9-*N*-(biphenyl-4-carbonyl)-amino-9-deoxy-Neu5Ac (BPC-Neu5Ac) and PROP (2-benzyl-Neu5NPro) compounds bound to Siglec-1 (55, 56) as templates, and generated a model of GSC839 bound to CD22 in such a way that the benzyl and the biphenyl groups of GSC 839 correspond to the benzyl group of PROP and the biphenyl group of BPC-Neu5Ac, respectively. It is also taken into account that GSC839 contains an additional carbon chain between the C9 position of sialic acid and the biphenyl group and a hydroxy group at the tip of the biphenyl group compared to BPC-Neu5Ac. The homology model of GSC839-bound mouse CD22 reveals favorable interactions between the aromatic moieties of both the benzyl and biphenyl groups of GSC839 and Phe68 and Tyr66, respectively (Figure 8B). Both residues are able to form π–π interactions and contribute to the high affinity of GSC839 to mouse CD22.

## Discussion

Here, we demonstrated that the synthetic sialoside GSC718 and GSC839 bind to both human and mouse recombinant CD22 proteins with high affinity (IC_50_ ~100 nM). These sialosides bind to CD22 expressed on the cell surface as well because we recently demonstrated using proximity labeling that GSC718 inhibits association of cell surface CD22 with its glycan cis-ligands (35). Both GSC718 and GSC839 modulate *in vitro* activation of mouse B cells and enhance antibody production in mice. These sialosides do not regulate activation of CD22^−/−^ B cells or enhance antibody production in CD22^−/−^ mice, suggesting that these sialosides specifically regulate CD22. Treatment with these synthetic sialosides down-modulates B cell proliferation induced by BCR ligation, whereas the same treatment does not alter BCR ligation-induced proliferation of ST6GalI^−/−^ B cells, suggesting that this effect of the sialosides depends on endogenous CD22 ligands. Because CD22 ligands are suggested to augment BCR signaling by inhibiting CD22 function (30–33), GSC718 and GSC839 appear to down-modulate BCR ligation-induced B cell proliferation by reversing ligand-mediated regulation on CD22. By contrast, both GSC718 and GSC839 augment proliferative responses to TLR ligands and CD40 ligation *in vitro* in WT but not CD22^−/−^ B cells. CD22 inhibits proliferative responses to TLR ligands and CD40 ligation (33, 47–49) as well as BCR ligation-induced signaling (24, 25), although it is not yet clear how CD22 regulates signaling through CD40 and TLRs. Therefore, GSC718 and GSC839 appear to suppress CD22 thereby enhancing B cell responses to TLR ligands and CD40 ligation. Because these sialosides enhance proliferative responses to TLR ligands in ST6GalI^−/−^ as well as WT B cells, CD22 ligands are not required for this effect of the sialosides. These sialosides also enhance proliferation of anti-CD40-stimulated ST6GalI^−/−^ B cells. Proliferation of ST6GalI^−/−^ B cells stimulated with anti-CD40 and sialosides are slightly less than that of WT B cells. There might be a minor involvement of α2,6 sialic acid in CD40-mediated B cell activation although the mechanism is not clear. Thus, GSC718 and GSC839 may directly inhibit CD22 by binding to CD22. Taken together GSC718 and GSC839 downregulate BCR ligation-induced B cell activation by reversing ligand-mediated regulation on CD22, but augment B cell activation by TLR ligands or CD40 ligation through direct inhibition of CD22. It is not yet clear how CD22-binding sialosides regulate CD22 differentially in the presence or absence of BCR ligation. One possible mechanism may be that BCR ligation activates CD22 ligands to regulate CD22, thereby overwhelming the direct inhibition of CD22 induced by CD22-binding sialosides.

Here, we demonstrated that GSC839 augments proliferation of B cells stimulated with transient BCR ligation and continuous CD40 ligation. With these stimuli, B cell proliferation depends on both BCR and CD40 signaling as is the case for *in vivo* B cell responses to antigens (51). Although B cell proliferation induced by continuous BCR ligation is reduced by GSC839, GSC839 augments B cell proliferation induced by transient BCR ligation with or without anti-CD40. GSC839 might differentially regulate CD22-mediated signal inhibition either between early and late phases of the culture or between BCR signaling and signaling induced by other stimuli such as CD40 ligation or mitogenic factors in the culture though mechanisms for the differential regulation is not yet clear. Moreover, we demonstrated that GSC718 and GSC839 augment B cell response to TLR ligands and CD40 ligation. CD40 signaling is crucial for T cell-dependent B cell activation (57) and enhances plasma cell differentiation (58, 59). TLR signaling in B cells is shown to be important for antibody response (60–64). Thus, augmented proliferation induced by the combination of BCR ligation and CD40 ligation, and enhanced reactivity to CD40 ligation and TLR ligands may account for the adjuvant activity of GSC839.

Sialic acid-containing antigens suppress B cell activation and antibody production by interacting with both BCR and CD22, thereby enhancing CD22-mediated signal inhibition (28, 65). However, CD22 appears to suppress B cell responses to non-sialylated antigens because CD22 down-modulates BCR signaling induced by F(ab′)_2_ fragments of anti-IgM devoid of glycosylation(37, 66–68). Thus, reversal of CD22-mediated signal inhibition augments B cell activation to both sialylated and non-sialylated antigens though B cell response to sialylated antigens may be more strongly augmented by CD22 inhibition. Here, we demonstrate that GSC839 augments antibody responses to OVA, which is not sialylated (69). Because CD22 suppresses B cell response to both sialylated and non-sialylated antigens, GSC839 augments antibody responses to OVA by reversing CD22-mediated signal inhibition.

Although various compounds have been isolated that enhance immune responses, most of them augment immune response by activating pattern recognition receptors (PRR) such as TLRs (70). PRR ligands activate DCs crucial for T cell activation, but also activate inflammatory cells such as macrophages and neutrophils. By contrast, both GSC718 and GSC839 bind to CD22 expressed in B cells and DCs but not macrophages or neutrophils. GSC718 does not activate DCs probably because of low CD22 expression in these cells. *In vivo* treatment of GSC839 does not induce production of inflammatory cytokines or induce recruitment of inflammatory cells, whereas CpG and alum induce production of inflammatory cytokines and recruitment of inflammatory cells, respectively. Thus, CD22-binding sialosides do not induce inflammatory responses probably because it specifically activates B cells. Lack of inflammation may lead to preferential IgG1 production induced by GSC839. Moreover, CD22^−/−^ mice do not develop either inflammatory or autoimmune diseases. This finding supports the safety of GSC839 because GSC839 appears to augment antibody production by suppressing CD22. Here, we failed to demonstrate how these sialosides enhance antibody production *in vivo* because the adjuvant effect of these sialosides was clear when subcutaneously immunized with a small amount (2.5 µg) but not a larger amount (10 µg) of the antigen (OVA). Immunization with the small amount of the antigen induces antibody production but did not induce measurable changes in immune cells. With a larger amount of the antigen, B cell activation may depend more on BCR signaling, which is down-modulated by CD22-binding sialosides. When immunized with a small amount of antigen, GSC839 enhances specific antibody production by itself as efficiently as CpG oligo that is now under clinical trial as a vaccine adjuvant for human (71). Thus, CD22-binding sialosides that enhance specific antibody production without inducing inflammation may be a good candidate for a safe adjuvant for human.

Only a small fraction of the proteins involved in diseases can be regulated by small chemical compounds. Druggability was addressed initially by homology to the proteins that are regulated by chemical compounds, but later by the presence of molecular pockets that can accommodate chemical compounds (72, 73). Here, we generated a molecular model of mouse CD22 complexed with GSC839, and demonstrated that aromatic moieties in GSC839 may form π–π interactions with the aromatic amino acid residues Phe68 and Tyr66 in CD22 without involvement of a molecular pocket. Previously, Zaccai et al. generated a model of human CD22 complexed with BPC-Nue5Ac, and suggested that the biphenyl group of BPC-Neu5Ac is sandwiched by Met129 and Arg131 (55). The guanidyl group of Arg131 may form a cation–π interaction to the biphenyl group of GSC839. Arg131 in human CD22, and Tyr66 in mouse CD22, the key residues for interaction with the biphenyl groups, correspond to Pro141 in mouse CD22 and Pro62 in human CD22, respectively. Both of these prolines may not contribute to the affinity to the biphenyl group. Thus, human and mouse CD22 interact with the biphenyl groups of BPC-Neu5Ac and GSC839, respectively, with similar but distinct mechanisms, although GSC839 binds to human and mouse CD22 with comparable affinity.

GSC718 was the first synthetic ligand for both human and mouse CD22 with nanomolar potency (6). Later, Prescher et al. developed a C2/C4/C9-modified sialoside that binds to human CD22 with higher affinity (2 nM) (8). However, selectivity to CD22 of this sialoside has not yet been known. Rillahan et al. developed (200 nM) a C2/C5/C9-modified sialoside that selectively binds to human CD22 with the affinity comparable to or slightly less than that of GSC718 and GSC839 (9). Here, we demonstrated that CD22 is required for GSC839 to regulate antibody production, and GSC839 does not induce production of inflammatory cytokines or recruit inflammatory cells. Thus, it is unlikely that GSC839 regulate other Siglecs than CD22. This is in agreement with our finding that GSC839 and GSC718 inhibit binding of recombinant CD22 but not other mouse Siglecs to cell surface ligands (Akatsu et al., manuscript in preparation), indicating that these sialosides selectively bind to CD22. Thus, GSC718 and GSC839 show the highest affinity to CD22 as a selective CD22 ligand so far developed. High-affinity CD22 ligands have been best developed as synthetic Siglec ligands. Synthetic ligands for other Siglecs still do not achieve IC50 of less than 100 nM (16).

In summary, synthetic sialosides that bind to mouse CD22 with high-affinity by forming π–π interactions with CD22 regulate B cell activation *in vitro* and augment antibody production in mice. To our knowledge, this is the first example of monomeric high-affinity synthetic Siglec ligand that regulates immune responses *in vivo*. Our results suggest that development of the monomeric Siglec ligands that regulate other members of Siglecs may also be possible even if they do not possess druggable pockets. Most of the Siglecs carry signaling function, and each member of the Siglec family is expressed in specific immune cell types. Thus, monomeric Siglec ligands that regulate distinct Siglecs may become unique compounds that contain immune regulatory activity although multimers of Siglec ligands and nanoparticles containing Siglec ligands have already been shown to possess therapeutic potentials by targeting various molecules to Siglecs.

## Ethics Statement

All mice used in this study were bred and maintained in a specific pathogen-free animal facility of Tokyo Medical and Dental University. All procedures followed the guidelines of Tokyo Medical and Dental University for animal research and were approved by Institutional Animal Care and Use Committee, Tokyo Medical and Dental University.

## Author Contributions

NM, TY, CA, and HY performed experiments and analyzed data. AI, AU, NW, HA-A, HA, HI, and MK designed and synthesized sialosides. HT, SK, TFT, and JM prepared and provided materials. NN and NI did homology modeling. TT designed the study, analyzed data, and wrote the manuscript.

## Conflict of Interest Statement

The authors declare that the research was conducted in the absence of any commercial or financial relationships that could be construed as a potential conflict of interest. The reviewer NR and handling Editor declared their shared affiliation.
