# Supplementary material for: CD22-Binding Synthetic Sialosides Regulate B Lymphocyte Proliferation Through CD22 Ligand-Dependent and Independent Pathways, and Enhance Antibody Production in Mice
Source: Front Immunol. 2018 Apr 19;9:820. doi: 10.3389/fimmu.2018.00820 (PMC5917077; doi:10.3389/fimmu.2018.00820)
Supplement: Supplementary file 1 [file Presentation_1.PDF]

Supplementary Material

CD22-binding synthetic sialosides regulate B lymphocyte proliferation through cis-ligand-dependent and independent pathways, and enhances antibody production in mice

Naoko Matsubara, Akihiro Imamura, Tatsuya Yonemizu, Chizuru Akatsu, Hongrui Yang, Akiharu Ueki, Natsuki Watanabe, Hajjaj Abdu-Allah, Nobutaka Numoto, Hiromu Takematsu, Shinobu Kitazume, Thomas F Tedder, Jamey D Marth, Nobutoshi Ito, Hiromune Ando, Hideharu Ishida, Makoto Kiso, and Takeshi Tsubata*

*** Correspondence:** Takeshi Tsubata: tsubata.imm@mri.tmd.ac.jp

1 Supplementary Materials and Methods

Synthesis of GSC839

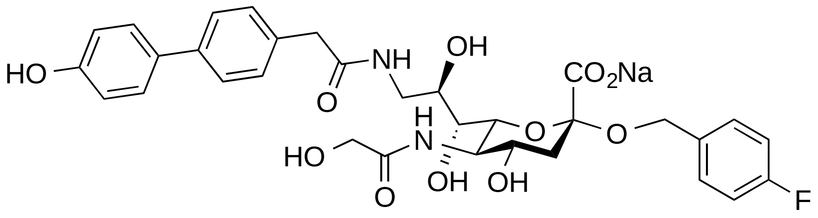

GSC839

GSC839 was synthesized according to the following synthetic procedures.

*4-Fluorobenzyl 5-amino-9-azido-3,5,9-trideoxy-*D*-glycero-α-*D*-galacto-2-nonulopyranosidono-1,5-lactam*

A mixture of methyl (phenyl 4,7,8-tri-*O*-acetyl-9-azido-2-thio-3,5,9-trideoxy-5-trifluoroacetamido-D-*glycero*-β-D-*galacto*-2-nonulopyranosid)onate (500 mg, 0.81 mmol) and 4-fluorobenzyl alcohol (87 µg, 0.81 mmol) in propionitrile (4.1 mL), which was distilled prior to use, was transferred into a two-neck round bottom flask, in which *N*-iodosuccinimide (270 mg, 1.20 mmol) and 3Å molecular sieves (324 mg) were placed, using a cannula and the suspension was stirred for 30 min at rt. The mixture was cooled to -30 °C and the stirring was continued for 30 min. Subsequently, triethylsilyl trifluoromethanesulfonate (54 µL, 0.24 mmol) was added to the mixture at -30 °C. After stirring for 1 h at the same temperature as the reaction was monitored by TLC (Toluene/EtOAc = 4:1), the reaction

was quenched by the addition of satd aq Na_2CO_3 at rt and the stirring was continued for 10 min. The precipitate was filtered through Celite pad and the pad was washed with chloroform. The combined filtrate and washings were washed with satd aq $Na_2S_2O_3$, dried over Na_2SO_4, and concentrated. The resulting residue was roughly purified by gel filtration column chromatography (LH-20, $CHCl_3$/MeOH = 1:1) to give the desired 4-fluorobenzyl glycosides as a mixture of stereoisomers. The obtained glycosides were then dissolved in methanol (81 mL). To the mixture was added Drierite (1.00 g) and the suspension was stirred for 1 h at rt. Subsequently, sodium methoxide (28% solution in MeOH, 386 mg, 2.5 mmol) was added to the mixture. After stirring for 72 h at reflux as the reaction was monitored by TLC ($CHCl_3$/MeOH = 5:1), the reaction was quenched by Dowex-50 (H^+) resin. After the resin was filtered off, the combined filtrate and washings were concentrated. The resulting residue was purified by flash silica gel column chromatography ($CHCl_3$/MeOH = 15:1) to give the desired lactamized product (189 mg, 61%) as a slightly yellow foamy compound.

4-Fluorobenzyl 4,7,8-tri-O-acetyl-5-amino-9-azido-3,5,9-trideoxy-D-glycero-α-D-galacto-2-nonulopyranosidono-1,5-lactam

To a solution of 4-Fluorobenzyl 5-amino-9-azido-3,5,9-trideoxy-D-*glycero*-α-D-*galacto*-2-nonulopyranosidono-1,5-lactam (189 mg, 0.49 mmol) in pyridine (2.5 mL) were added acetic anhydride (0.93 mL, 9.8 mmol) and 4-dimethylaminopyridine (12 mg, 0.10 mmol) at room temperature. After stirring for 1 h at rt as the reaction was monitored by TLC ($CHCl_3$/MeOH = 30:1), the reaction was quenched by the addition of dry methanol and then co-evaporated with toluene. The residue was diluted with $CHCl_3$, washed with 2 M HCl, H_2O, and satd aq $NaHCO_3$, dried over Na_2SO_4, concentrated. The resulting residue was exposed to high vacuum for 1 h and then dissolved in tetrahydrofuran (9.8 mL). To the solution was added hydrazine acetate (90 mg, 0.98 mmol) at rt. After stirring for 2 h at rt as the reaction was monitored by TLC ($CHCl_3$/MeOH = 20:1), the reaction mixture was diluted with ethyl acetate and washed with 2 M HCl, H_2O, satd aq $NaHCO_3$, and brine, dried over Na_2SO_4, concentrated. The resulting residue was purified by flash silica gel column chromatography (EtOAc/*n*-Hexane= 2:3) to give the desired product (218 mg, 87%) as a white foamy compound.

4-Fluorobenzyl 4,7,8-tri-O-acetyl-9-azido-5-tert-butoxycarbonyl-3,5,9-trideoxy-D-glycero-α-D-galacto-2-nonulopyranosidono-1,5-lactam

To a solution of 4-Fluorobenzyl 4,7,8-tri-*O*-acetyl-5-amino-9-azido-3,5,9-trideoxy-D-*glycero*-α-D-*galacto*-2-nonulopyranosidono-1,5-lactam (218 mg, 0.43 mmol) in acetonitrile (8.6 mL) were added di-*tert*-butyl dicarbonate (142 μL, 0.65 mmol) and DMAP (5.3 mg, 43 μmol) at rt. After stirring for 10 min at rt as the reaction was monitored by TLC (EtOAc/*n*-Hexane= 2:3), the reaction mixture was diluted with chloroform and washed with 2 M HCl, H_2O, and satd aq $NaHCO_3$, dried over Na_2SO_4, concentrated. The resulting residue was purified by flash silica gel column chromatography (EtOAc/*n*-Hexane= 1:1) to give the desired product (252 mg, 96%) as a white foamy compound.

Methyl (4-fluorobenzyl 9-azido-5-tert-butoxycarbamoyl-3,5,9-trideoxy-D-glycero-α-D-galacto-2-nonulopyranosid)onate

To a solution of 4-fluorobenzyl 4,7,8-tri-*O*-acetyl-9-azido-5-*tert*-butoxycarbonyl-3,5,9-trideoxy-D-*glycero*-α-D-*galacto*-2-nonulopyranosidono-1,5-lactam (252 mg, 0.41 mmol) in methanol (41 mL) was added sodium methoxide (28% solution in MeOH, 791 mg, 4.1 mmol) at rt. After stirring for 1 h at rt as the reaction was monitored by TLC ($CHCl_3$/MeOH = 15:1), the reaction was quenched by

Dowex-50 (H^+) resin. After the resin was filtered off, the combined filtrate and washings were concentrated. The resulting residue was purified by flash silica gel column chromatography (EtOAc/*n*-Hexane= 2:3) to give the desired product (200 mg, 95%) as a white foamy compound.

Methyl {4-fluorobenzyl 5-tert-butoxycarbamoyl-9-(4'-O-tert-butyldimethylsilyl-4-biphenyl)acetamido-3,5,9-trideoxy-D-glycero-α-D-galacto-2-nonulopuranosid}onate

To a solution of methyl (4-fluorobenzyl 9-azido-5-*tert*-butoxycarbamoyl-3,5,9-trideoxy-D-*glycero*-α-D-*galacto*-2-nonulopyranosid)onate (100 mg, 0.19 mmol) in tetrahydrofuran/water (1.9 mL, 10:1) was added triphenylphosphine (249 mg, 0.95 mmol) at rt. After stirring for 22 h at rt as the reaction was monitored by TLC ($CHCl_3$/MeOH = 8:1), 4'-*O*-*tert*-butyldimethylsilyl-4-biphenylacetic acid succinimidyl ester (101 mg, 0.23 mmol) was added to the reaction mixture at rt. After stirring for 1 h at rt as the reaction was monitored by TLC ($CHCl_3$/MeOH = 8:1), the reaction mixture was diluted with ethyl acetate and washed with H_2O and brine, dried over Na_2SO_4, concentrated. The resulting residue was purified by flash silica gel column chromatography ($CHCl_3$/MeOH = 60:1 $\rightarrow$ 40:1) to give the desired product (133 mg, 86%) as a white foamy compound.

Methyl {4-fluorobenzyl 5-acetoxyacetamido-9-(4'-O-tert-butyldimethylsilyl-4-biphenyl)acetamido-3,5,9-trideoxy-D-glycero-α-D-galacto-2-nonulopuranosid}onate

To a solution of methyl {4-fluorobenzyl 5-*tert*-butoxycarbamoyl-9-(4'-*O*-*tert*-butyldimethylsilyl-4-biphenyl)acetamido-3,5,9-trideoxy-D-*glycero*-α-D-*galacto*-2-nonulopuranosid}onate (133 mg, 0.16 mmol) in dichloromethane (2.4 mL) were added trifluoroacetic acid (0.6 mL) and anisole (35 µL, 0.16 mmol) at 0 °C. After stirring for 1 h at rt as the reaction was monitored by TLC ($CHCl_3$/MeOH = 7:1), the reaction mixture was co-evaporated with toluene. The residue was exposed to high vacuum for 1 h and then dissolved in *N*,*N*-dimethylformamide (3.2 mL). To the solution were added acetoxyacetic acid succinimidyl ester (69 mg, 0. 32 mmol) and triethylamine (44 µL, 0.32 mmol) at 0 °C. After stirring for 1.5 h at rt as the reaction was monitored by TLC ($CHCl_3$/MeOH = 7:1), the reaction mixture was co-evaporated with toluene. The residue was diluted with ethyl acetate and washed with H_2O and brine, dried over Na_2SO_4, concentrated. The resulting residue was purified by flash silica gel column chromatography ($CHCl_3$/MeOH = 40:1 $\rightarrow$ 30:1) to give the desired product (106 mg, 81%) as a white foamy compound.

Sodium {4-fluorobenzyl 5-hydroxyacetamido-9-(4'-hydroxy-4-biphenyl)acetamido-3,5,9-trideoxy-D-glycero-α-D-galacto-2-nonulopuranosid}onate (GSC839)

To a solution of methyl {4-fluorobenzyl 5-acetoxyacetamido-9-(4'-*O*-*tert*-butyldimethylsilyl-4-biphenyl)acetamido-3,5,9-trideoxy-D-*glycero*-α-D-*galacto*-2-nonulopuranosid}onate (49.0 mg, 60 µmol) in tetrahydrofuran (1.5 mL) was added 0.5 M aq sodium hydroxide (1.5 mL) at rt. After stirring for 1 h at rt as the reaction was monitored by TLC ($CHCl_3$/MeOH/H_2O = 6:3:0.5), the reaction mixture was evaporated. The resulting residue was purified by gel filtration column chromatography ($CHCl_3$/MeOH/H_2O = 5:4:1) to give GSC839 (37 mg, quantitative) as a white powder compound.
